# Genetic diversity and population structure of the Tibetan poplar (*Populus szechuanica var. tibetica*) along an altitude gradient

**DOI:** 10.1186/1471-2156-15-S1-S11

**Published:** 2014-06-20

**Authors:** Dengfeng Shen, Wenhao Bo, Fang Xu, Rongling Wu

**Affiliations:** 1 Center for Computational Biology, National Engineering Laboratory for Tree Breeding, Key Laboratory of Genetics and Breeding in Forest Trees and Ornamental Plants, Ministry of Education, College of Biological Sciences and Biotechnology, Beijing Forestry University, Beijing, China

**Keywords:** Gene flow, Microsatellite, Genetic diversity, Qinghai-Tibet plateau, *Populus szechuanica var. tibetica*.

## Abstract

**Background:**

The Tibetan poplar (*Populus szechuanica var. tibetica *Schneid), which is distributed at altitudes of 2,000-4,500 m above sea level, is an ecologically important species of the Qinghai-Tibet Plateau and adjacent areas. However, the genetic adaptations responsible for its ability to cope with the harsh environment remain unknown.

**Results:**

In this study, a total of 24 expressed sequence tag microsatellite (EST-SSR) markers were used to evaluate the genetic diversity and population structure of Tibetan poplars along an altitude gradient. The 172 individuals were of genotypes from low-, medium- and high-altitude populations, and 126 alleles were identified. The expected heterozygosity (*H*_E_) value ranged from 0.475 to 0.488 with the highest value found in low-altitude populations and the lowest in high-altitude populations. Genetic variation was low among populations, indicating a limited influence of altitude on microsatellite variation. Low genetic differentiation and high levels of gene flow were detected both between and within the populations along the altitude gradient. An analysis of molecular variance (AMOVA) showed that 6.38% of the total molecular variance was attributed to diversity between populations, while 93.62% variance was associated with differences within populations. There was no clear correlation between genetic variation and altitude, and a Mantel test between genetic distance and altitude resulted in a coefficient of association of r = 0.001, indicating virtually no correlation.

**Conclusion:**

Microsatellite genotyping results showing genetic diversity and low differentiation suggest that extensive gene flow may have counteracted local adaptations imposed by differences in altitude. The genetic analyses carried out in this study provide new insight for conservation and optimization of future arboriculture.

## Introduction

Altitude gradients represent one of the most useful natural environments to investigate ecological and evolutionary responses of biota to geophysical influences [1]. For species from habitats which cover different altitudes, differences in their spatial population structure could be due to restricted gene movement, as a result of non-random mating or geographic barriers [2,3]. Outliers of species found at the boundaries of their distribution zones could be subject to limited gene flow, a small population size and founder effects, all of which lead to a decrease in genetic diversity and an increase in population differentiation [4]. For species living in mountainous areas, altitude changes represent a series of physical factors that can result in the establishment of different populations and species. These factors form barriers, which influence genetic diversity and population structure [5-7], and include factors such as rainfall [8] and temperature [9]. There is no general rule to summarize the relationship between genetic diversity and altitude; for trees on mountainsides, the pattern of genetic diversity along the altitude gradient is divided into four groups. (1) Populations at an intermediate altitude have greater diversity than populations at lower and higher altitudes, due to local adaptation and milder environmental conditions [10,11]. (2) Populations at higher altitudes have greater diversity than those at lower altitudes if the higher altitude conditions are similar to their home sites, representing higher fitness [12]. (3) Populations at lower altitudes have greater diversity than those at higher altitudes, as higher altitudes impede growth and the expanding of species countering the bottleneck leaded to decrease of genetic diversity [13]. (4) Populations show no differences in diversity at differing altitudes [14], the pattern may be due to that the sampling area was part of main distribution area, limited number of populations sampled along the gradient may cause the failure to detect altitude-related trends. On the other hand, if the sampled population was large enough, extensive gene flow and other factors also could lead to the similar pattern..

The Qinghai-Tibetan Plateau (QTP) is the highest and largest plateau in the world, with a mean altitude of 4 000 m above sea level, and an area of 2.5 × 10^6 ^km^2^. In recent years, the QTP has become a hotspot for plant phylogeographical studies [15,16], focusing mainly on the population dynamics that took place during the Quaternary (reviewed in Qiu *et al*.) [17]. However, genetic variation patterns along altitudinal gradients of the QTP remain unclear.

The Tibetan poplar belongs to *Populus *sect. *Tacamahaca *in the genus *Populus *and is an ecologically important species, mainly distributed in Sichuan and Tibet at altitudes from 2 000 to 4 500 m [18]. Recent studies have focused mainly on the phylogenic and physiological mechanisms responsible for its resistance to the harsh environment where the lowest temperature is -30°C and the annual average temperature is between 4°C to 12°C [19]. However, there is a pressing need to understand the genetic diversity along altitude gradients. In this paper we investigated the genetic variation of the Tibetan poplar along an altitude gradient using microsatellite genotyping. The specific objectives were: (1) to understand the genetic variation and differentiation within and between populations, and (2) to detect any influence of altitude gradients on genetic diversity.

In this study, a total of 24 EST- SSR loci based on *Populus euphratica *transcriptome [20] were used to analyze the genetic diversity and population structure of Tibetan poplar populations at different altitudes in the Sejila mountain area. The objectives were to provide a complete picture of the genetic diversity of Tibetan poplar populations at different altitudes in the Sejila mountain area, and to identify a relationship between genetic variation and differences in altitude.

## Materials and methods

### Sampling strategy and DNA extraction

**Figure 1:**
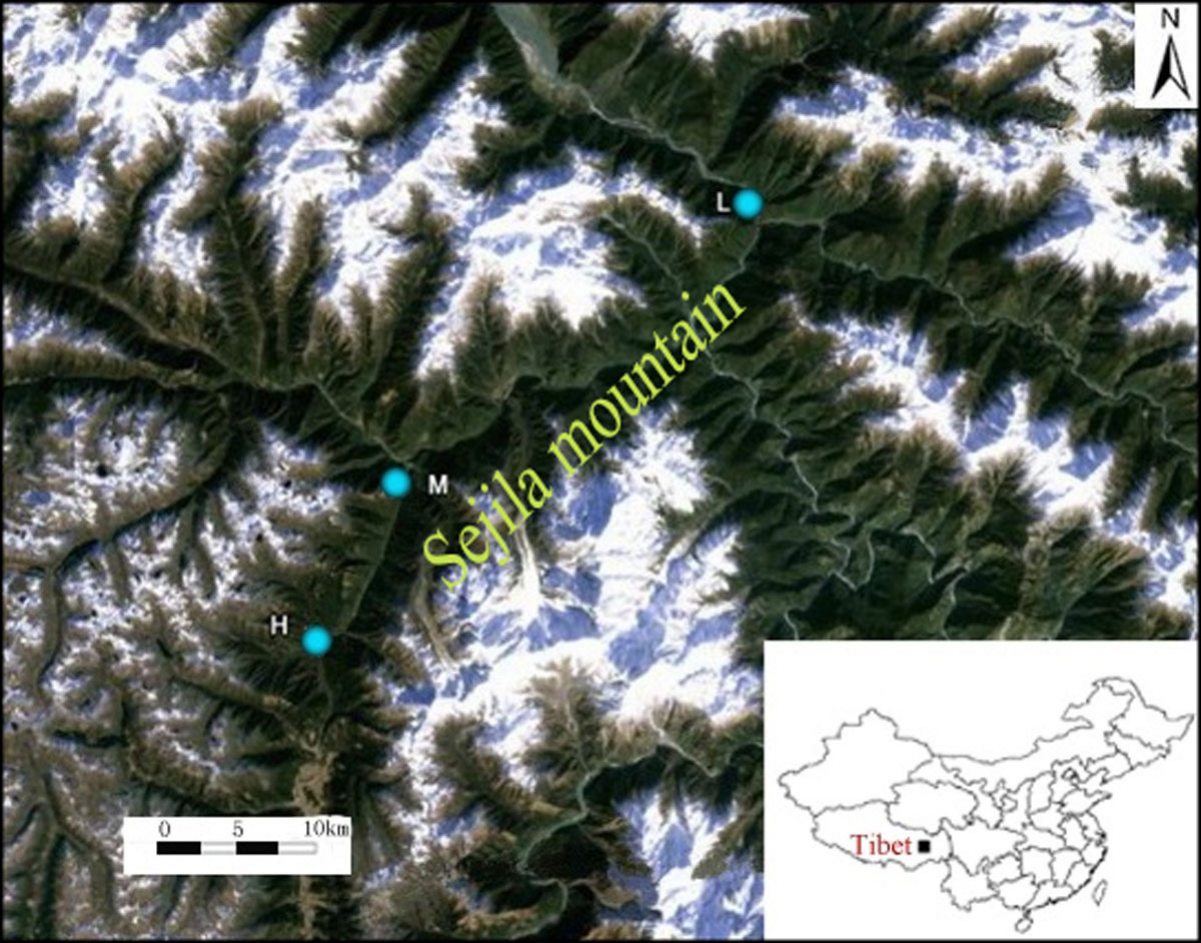
***Populus szechuanica *population locations**. In the Sejila mountain area, we selected three different altitude gradients to select samples.

We collected leaves from 64, 34 and 74 individuals from high-, medium- and low-altitude populations, respectively (Figure 1, Table 1). Our sampling scheme was to divide the distribution areas of the Tibetan poplar in the Sejila mountains (in southeastern Tibet) into three altitude-gradient groups (high, medium, and low), even though the trees are distributed continuously throughout the area. We selected individuals at a minimum of 30 m apart to prevent selection of clones. The leaf was rapidly dehydrated using silica gel beads. Total genomic DNA was extracted from approximately 0.5 g of silica-dried leaf using a modified version of the cetyltrimethyl ammonium bromide method [21]. The quality and concentration of the extracted DNA were determined by 1% agarose gel electrophoresis and ultraviolet spectrophotometry. The DNA samples were diluted to 5-10 ng/μL for use as the template for polymerase chain reaction (PCR) amplification.

**Table 1:** Locations of *Populus szechuanica *populations.

Pop code	Elevation(m)	Location	Latitude(N)	Longitude(E)	Sample size
Low	2000-2100	Tongmai, Linzhi, Tibet	30°05.81	95°04.15	64
Middle	2300-2700	Lulang, Linzhi, Tibet	30°10.33	94°56.15	34
High	2900-3090	Lulang, Linzhi, Tibet	29°50.9	94°45.8	74

### Primer selection

113 EST-SSR primer pairs based on the *Populus euphratica *transcriptome [20] were developed and tested for suitability in the Tibetan poplar. DNA extracted from four Tibetan poplar individuals was amplified, and the amplicons were sequenced to confirm the existence of and enumerate repeat motifs. DNA from eight individuals was used to test for polymorphisms of the successfully amplified primers. SSRs were selected if they had at least three alleles and exhibited robust amplification.

### SSR amplification

After screening, 24 primer pairs were selected for the PCR analysis. The forward primer of each pair was tagged with a section of the universal M13 sequence (5′-TGTAAAACGACGGCCAGT-3′) during synthesis. Each 10-μL PCR mixture contained 1× *Taq *buffer, 0.2 µM dNTPs, 10-20 ng template DNA, 1.6 pmol reverse primer, 1.6 pmol fluorescently labeled M13 primer, 0.4 pmol forward primer and 1 U *Taq *polymerase (BioMed). PCR amplification was performed using a Biometra thermocycler (Biometra, Goettingen, Germany) under the following conditions: 94°C for 5 min; 30 cycles of 94°C for 30 s, annealing at 56°C for 45 s and elongation at 72°C for 45 s; 8 cycles of 94°C for 30 s, annealing at 53°C for 45 s, elongation at 72°C for 45 s; and a final extension at 72°C for 10 min. The PCR products were separated by capillary electrophoresis using an ABI 3730xl DNA Analyzer (Applied Biosystems, Foster City, CA, USA) after confirmation of amplification on a 1.5% agarose gel. Approximately 0.5 μL of the PCR products obtained using each of the four fluorescently labeled primers was then combined. The products were separated using an ABI 3730xl DNA Analyzer with GeneScan-500 LIZ as an internal marker (Applied Biosystems). The amplicon fragments were sized using GeneMarker version 1.75 (Soft Genetics LLC, State College, PA, USA).

### Data analysis

The FLEXIBIN software was used for automated binning of the raw molecular data[22], and the Excel Microsatellite Toolkit [23] was used to convert the size data into a format suitable for further analysis. Genetic parameters were estimated under the hypothesis that all the loci were neutral, thereby presenting a true picture of the natural genetic structure affected by neutral forces such as genetic drift and gene flow, etc. There are several methods of investigating whether a particular locus has been under selection pressure. We performed the *F*_ST _outlier test using LOSITAN [24] to identify candidate SSR loci possibly under selection pressure [25]. After removal of outlier loci, the remaining data were used to estimate the genetic diversity of the population. Genetic diversity parameters used included: number of alleles (*N*a); observed heterozygosity (*H*_O_); expected heterozygosity (*H*_E_) within a subpopulation; Wright's fixation indices for within-subpopulation (*F*_IS_) and in the total population (*F*_IT_); and pair-wise differentiation among subpopulations (*F*_ST_), according to Weir & Cockerham [26]. *F_IS _*measures the deviation from the Hardy-Weinberg equilibrium (HWE) of genotype frequencies in sub-populations, whereas *F*_IT _measures the deviation from HWE in the total population. The values of *F*_IT _and *F*_IS _can be negative, whereas *F*_ST _is always a positive value. The Shannon's diversity index was conducted using Nei's model, along with the expected heterozygosity [27]. Gene flow (*N*m) was calculated to ascertain the conditions of gene communication among populations, and was estimated as follows: *Nm = (1- F_ST_)/4 F_ST _*[28]. Summary statistics were calculated using POPGENE version 1.32 [29]. Inter- and intra-population differentiation was determined by AMOVA analysis using the GenAlEx software version 6.41 [30]. Clustering, based on a Bayesian model which assumed that all the individuals were from K real populations (where K may be unknown), each of which is characterized by a set of allele frequencies at each locus, the method attempts to assign individuals to populations on the basis of their genotypes, while simultaneously estimating population allele frequencies. The model was used to evaluate the genetic structures of the Tibetan poplar populations using STRUCTURE in its extended version 2.3.3 [31,32]. STRUCTURE is based on a model-based clustering algorithm that applies a Bayesian framework and the Markov chain Monte Carlo (MCMC) algorithm. The optimum number of subpopulations (K) was confirmed after 20 independent runs for each value of K between 1 and 10. The length of the burn-in period and number of MCMC reps after burn-in were set to 25,000 and 100,000, respectively. The K subpopulations identified indicated clusters characterized by a set of allele frequencies at each locus, where individuals were assigned to a subpopulation, or to two or more populations, if the genotype indicated that they are admixed [33]. In this study, the identification of K used the model developed by Evanno *et al*. [34]. The Bayesian framework was not used to estimate the non-homogeneous original populations, instead we used ΔK, which was based on the rate of change in the log probability of data between successive Ks. STRUCTURE accurately detected the uppermost hierarchical level structure for the scenarios tested.

A Mantel test, performed with GenAlEx version 6.41 [30], was used to calculate the coefficient of association between genetic distance and altitude.

## Results

### SSR genotyping

**Figure 2:**
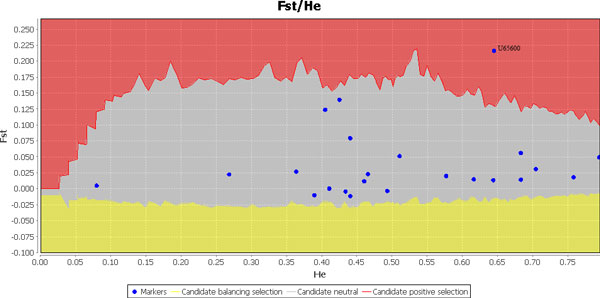
***F_ST _*values of 24 microsatellite loci vs heterozygosity in the *Populus szechuanica *population**. The dot in the red area is the locus U65600 which is positively under nature selection.

SSRs are generally used in genetic diversity studies as evolutionary neutral markers. In this study, 24 SSR primer pairs were developed using the *Populus euphratica *genome, which were transferable to the Tibetan poplar. Sequencing results were uploaded to GenBank, (Table 2). In total, 114 alleles for 24 loci were amplified (mean = 4.75, SD = 2.71), with locus 7 having 12 alleles and exhibiting the most variation. Locus 18 was detected using LOSITAN based on its *F*_ST _value(0.16) [24], which showed that it was under positive natural selection (p=0.01) (Figure 2). Subsequently, the sequence was processed using NCBI BLAST [35], and there was high homology with a protein (ID: XM_002311699.1) present in *Populus trichocarpa*. This implied that this SSR locus could be under selection pressure.

**Table 2:** Descriptions of and references for the 24 SSR loci analyzed.

ID	Motif	Forward primer	Reverse primer	Allele range	Genebank ID
U18679	(TGC)7	ACATCAGGTGGTCTTCCTCG	GCATGCTTAAGGCACGAGTT	401-413	KF501217
U21882	(GAT)6	GCGGACGGTCTTGATTACAT	TCTTTCGACCCTTTTAGCGA	305-329	KF501218
U22153	(AC)13	TTCCACAAGCCATACCACAA	CCACCTTTCGTAACCTTGGA	269-281	
U61645	(TG)13	AATGGTATAGCCGGCCTCTT	ACAGGAGAAGGGGGAGATGT	220-222	KF501226
U63239	(CTC)6	TTTGCTCTGTGAACGCAATC	AGCTTGTGGATTTGTCTGCG	202-205	KF501227
U78717	(GT)10	CTGGTATGGATGGATTTGGG	ATTAAGCCCAAGCCTTCACC	237-239	KF501231
U7452	(AC)11	CCCCCTCCTTACATCTTATGG	CTGGAAAGTGCATCTCCGAT	265-287	KF501215
U64059	(AG)10	TGTGCAATTGTGAGGTCAAT	GCAACCTAAATGACCACCTTG	295-315	KF501228
U7459	(CT)12	TCCTTCCTTCACGAAGCACT	GTGGGCAAGCTCTTTGAAAC	325-337	KF501216
U60914	(AG)11	TTGACCCCCAGTTCAGATTC	GGCAAATTCGCCCTAGAATTA	226-238	KF501225
U78	(GAGCTG)3	TGTCAGCTCTTCACCACCTG	CAGAAAGGGAGAACCCACAA	176-194	KF501212
U74541	(AC)10	GACCCACACCCACAAAAGAT	TCACATGAATTTGCTCGAGTG	217-221	KF501230
U4192	(AAAAAT)3	GCAGTGGAGAAGAAGCATCC	CGTTGCTTTCGCAGACAATA	298-313	KF501213
U16390	(TGGGGA)3	TGGAGTCCGAGGAAGAGAGA	TCGTCACTTTTGCAAGCATC	454-560	
U16	(GAG)9	GGAGGACCAGATAAGGGAGC	TGGGGTAAGCTGACTTGCTT	222-234	KF501211
U45275	(TTC)7	TGCAGTTTTAGGCCTCTTCC	CTGCAGAATTCCACATCCAA	181-193	KF501224
U35013	(CT)16	TTTCCAGGGACAGAACTTCG	GATGGGGTGAGAGAGGAACA	110-134	
U65600	(AG)14	GTCTTTGGTGGCTACACCGT	CTCAATCCTTTCCTTGGTGG	229-240	KF501229
U37186	(CA)10	TAACATGGCGAGTAGGGACC	GCCAAACAGACCTCGATCAT	399-403	KF501221
U6496	(TTCTT)5	GTAAACAAAGGGACCCCTCC	CCCAAATCCCCAATTATTCC	290-298	KF501214
U34847	(AG)14	TCTCCTCTCCTTTCCACCAA	AAAAAGCCCAAGGATCAGGT	182-199	KF501219
U38100	(GT)12	GCTGTGCTTGAGGATGATGA	CCCTAATCCCACCTCTTGAA	210-225	KF501223
U38010	(TTG)12	ACCACCTTCATGTTCTTGGC	CCGTTTCTTTCACTCCCAAA	309-337	KF501222
U35536	(AGAAGT)3	TGAAATTGGGTGGTGCAGTA	CTCTTCACCAAAACCCTCCA	269-275	KF501220

### Genetic diversity

Previous assessment of genetic diversity among three populations of Tibetan poplar was based on allelic variation observed at 23 neutral microsatellite loci. In this study, a mean of 3.71 alleles per locus were confirmed for all 23 loci in 172 Tibetan poplar individuals. *H*_O _and *H*_E _are important parameters for assessing genetic diversity of populations, and they ranged from 0.40-0.42 and 0.48-0.49, respectively. In the three populations, as the results were consistent with the Na, it indicated that genetic variation was not significant, and the populations were similar in all parameters. *F*_IS _presented a similar pattern in that no significant difference was detected among populations; it ranged from -0.85 to 0.56 (mean = 0.17, SD = 0.18), with only a moderate difference for loci from trees at low altitude. The *F*_ST _was estimated at each locus for all individuals, and ranged from 0.0004 to 0.16 (table 3), indicating that there was no evidence to support the hypothesis that the populations differed from each othe r.

**Table 3:** Comparisons of genetic diversity and differentiation among *Populus szechuanica *populations along altitude gradients.

	L					M					H					total		
**Locus**	***Na***	***I***	***H*o**	***H*e**	***F*is**	***Na***	***I***	***H*o**	***H*e**	***F*is**	***Na***	***I***	***H*o**	***H*e**	***F*is**	***F*it**	***F*st**	***N*m**

ssr1	2	0.086	0.034	0.033	-0.017	2	0.215	0.111	0.105	-0.059	3	0.219	0.100	0.096	-0.046	-0.035	0.012	20.945
ssr2	3	0.272	0.095	0.120	0.209	5	0.762	0.303	0.345	0.121	4	1.068	0.487	0.587	0.172	0.237	0.093	2.450
ssr3	4	1.238	0.566	0.676	0.163	4	1.052	0.654	0.605	-0.081	5	1.136	0.433	0.628	0.310	0.174	0.046	5.143
ssr4	2	0.667	0.419	0.475	0.116	2	0.656	0.303	0.463	0.345	2	0.693	0.420	0.500	0.159	0.238	0.042	5.766
ssr5	3	0.645	0.254	0.376	0.323	3	0.627	0.387	0.362	-0.069	3	0.828	0.500	0.480	-0.042	0.069	0.007	34.496
ssr6	2	0.650	0.569	0.457	-0.245	2	0.688	0.367	0.495	0.259	2	0.585	0.429	0.396	-0.084	0.011	0.023	10.542
ssr7	8	1.588	0.639	0.768	0.168	10	1.761	0.688	0.758	0.093	7	1.489	0.767	0.715	-0.073	0.104	0.040	5.936
ssr8	9	1.566	0.542	0.712	0.238	5	1.274	0.643	0.666	0.035	5	1.164	0.514	0.618	0.167	0.163	0.017	14.129
ssr9	5	0.882	0.167	0.528	0.684	2	0.627	0.240	0.435	0.449	2	0.570	0.279	0.382	0.269	0.499	0.017	14.483
ssr10	3	0.733	0.508	0.507	-0.002	2	0.460	0.276	0.285	0.033	3	0.495	0.310	0.293	-0.059	0.097	0.104	2.148
ssr11	4	1.031	0.516	0.583	0.116	4	1.154	0.552	0.646	0.146	5	1.165	0.414	0.654	0.366	0.226	0.017	14.936
ssr12	2	0.689	0.535	0.496	-0.077	2	0.605	0.517	0.414	-0.248	2	0.466	0.177	0.291	0.393	0.041	0.062	3.784
ssr13	4	1.002	0.500	0.528	0.053	4	0.875	0.483	0.531	0.091	5	1.178	0.623	0.618	-0.009	0.062	0.021	11.776
ssr14	2	0.674	0.446	0.481	0.071	2	0.693	0.546	0.500	-0.092	2	0.680	0.541	0.487	-0.112	-0.040	0.005	47.305
ssr15	3	0.574	0.180	0.299	0.397	4	0.512	0.107	0.229	0.532	3	0.442	0.167	0.249	0.329	0.428	0.023	10.830
ssr16	3	0.732	0.377	0.467	0.193	3	0.647	0.464	0.401	-0.157	3	0.643	0.458	0.423	-0.084	-0.002	0.005	53.991
ssr17	5	1.095	0.245	0.569	0.569	6	1.565	0.407	0.753	0.459	7	1.449	0.258	0.700	0.632	0.563	0.029	8.384
ssr19	2	0.641	0.340	0.449	0.243	2	0.628	0.357	0.436	0.181	2	0.627	0.443	0.435	-0.018	0.137	0.000	675.978
ssr20	2	0.641	0.340	0.449	0.243	2	0.628	0.357	0.436	0.181	2	0.627	0.443	0.435	-0.018	0.137	0.000	675.978
ssr21	7	1.074	0.469	0.501	0.064	5	0.713	0.367	0.323	-0.136	6	0.497	0.250	0.226	-0.107	-0.008	0.025	9.730
ssr22	5	1.236	0.516	0.644	0.198	4	1.098	0.448	0.599	0.252	4	0.974	0.400	0.557	0.281	0.255	0.018	14.047
ssr23	5	1.286	0.661	0.692	0.045	5	1.424	0.759	0.731	-0.038	8	1.644	0.813	0.783	-0.037	0.008	0.020	12.438
ssr24	2	0.576	0.424	0.387	-0.094	2	0.562	0.433	0.375	-0.156	2	0.593	0.409	0.404	-0.014	-0.085	0.001	314.685
Mean	3.7917	0.856	0.404	0.488	0.172	3.583	0.840	0.422	0.477	0.115	3.833	0.836	0.407	0.475	0.143	0.172	0.034	7.020
St. Dev	1.9777	0.366	0.170	0.169	0.200	1.886	0.374	0.166	0.167	0.212	1.857	0.374	0.180	0.171	0.230	0.181	0.038	189.235

*Na*: allele number

*I*: Shannon index

*Ho*: observed heterozygosity

*He*: expected heterozygosity

*Fis*: fixation index in subpopulations

*Fit*: fixation index in total population

*Fst*: genetic differentiation of subpopulations

*Nm*: Gene flow estimated from Fst

### Genetic structure

The AMOVA indicated different levels of genetic variance among populations and among individuals within populations. Of the total genetic variance, 6.67% was ascribed to population divergence; the remainder was ascribed to the differences between individuals. However, there was a significant difference among populations (p < 0.001). In populations sampled from high, medium, and low altitudes, all genetic diversity parameters were similar, indicating no local adaptation or population differentiation in the study area.

**Figure 3:**
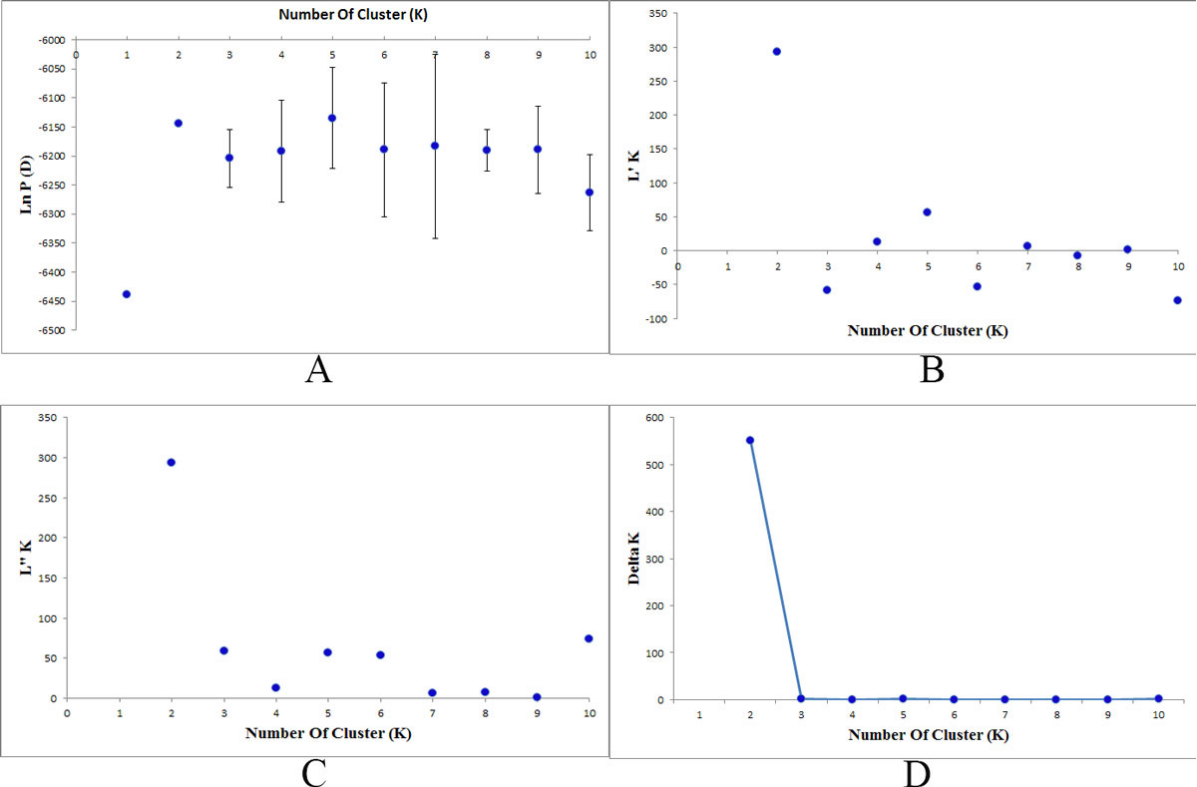
**Identification of K. the method of delta K was used to identify the accurate sub-clusters in the population**. In this population there is a peak of delta K in K = 2, the population is possibly composed of two sub-clusters.

**Figure 4:**
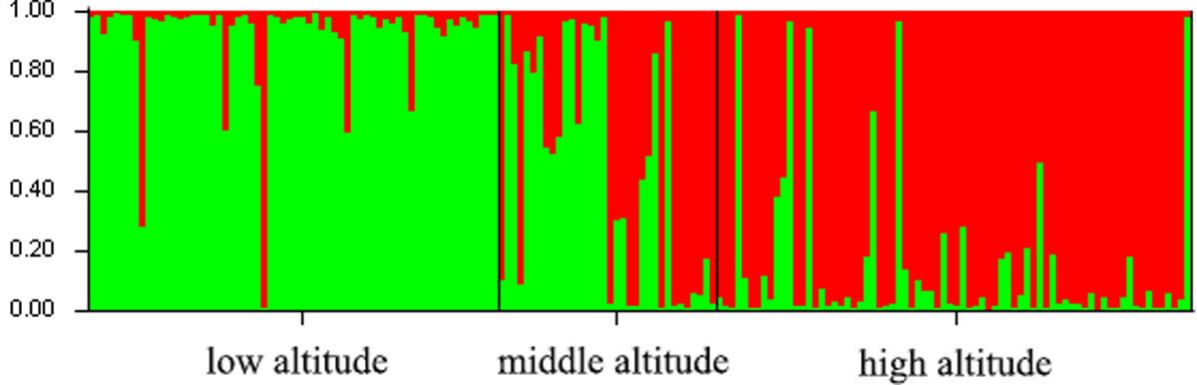
**Population structure of *Populus szechuanica *estimated by STRUCTURE**. In the figure, the individuals were sorted as the altitude of sampling distributed area.

**Figure 5:**
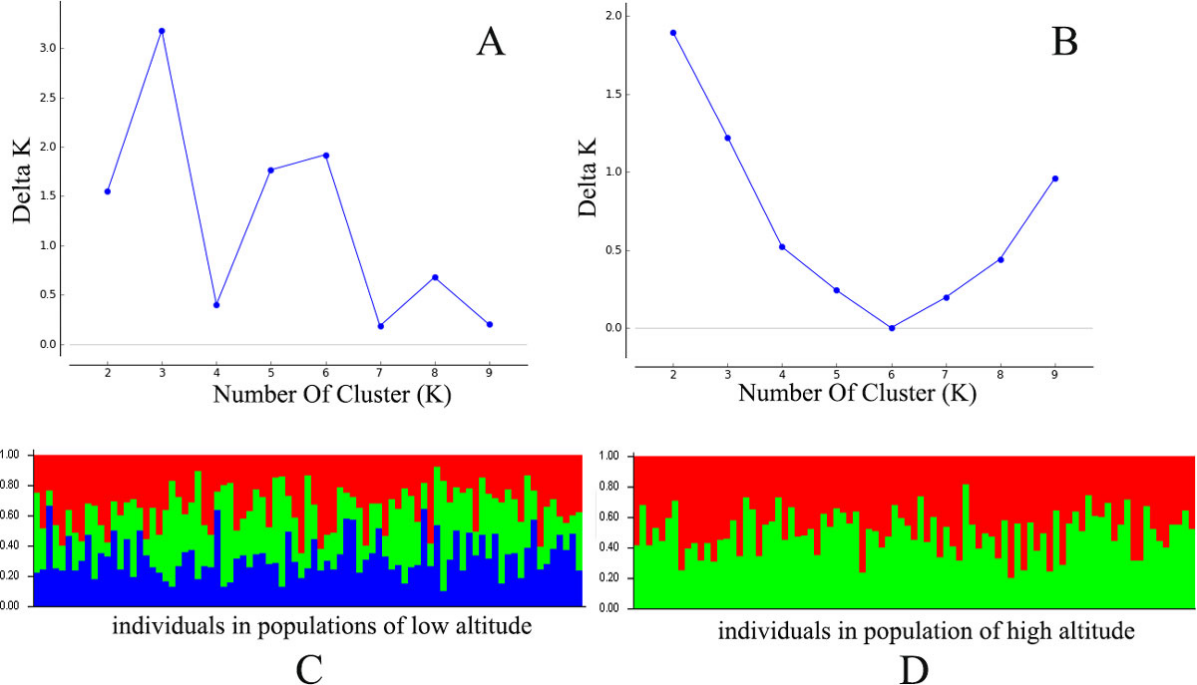
**Structure analysis of two sub-clusters**. A,C) the sub-clusters detected at low altitude; B,D) the sub-clusters detected at high altitude

**Figure 6:**
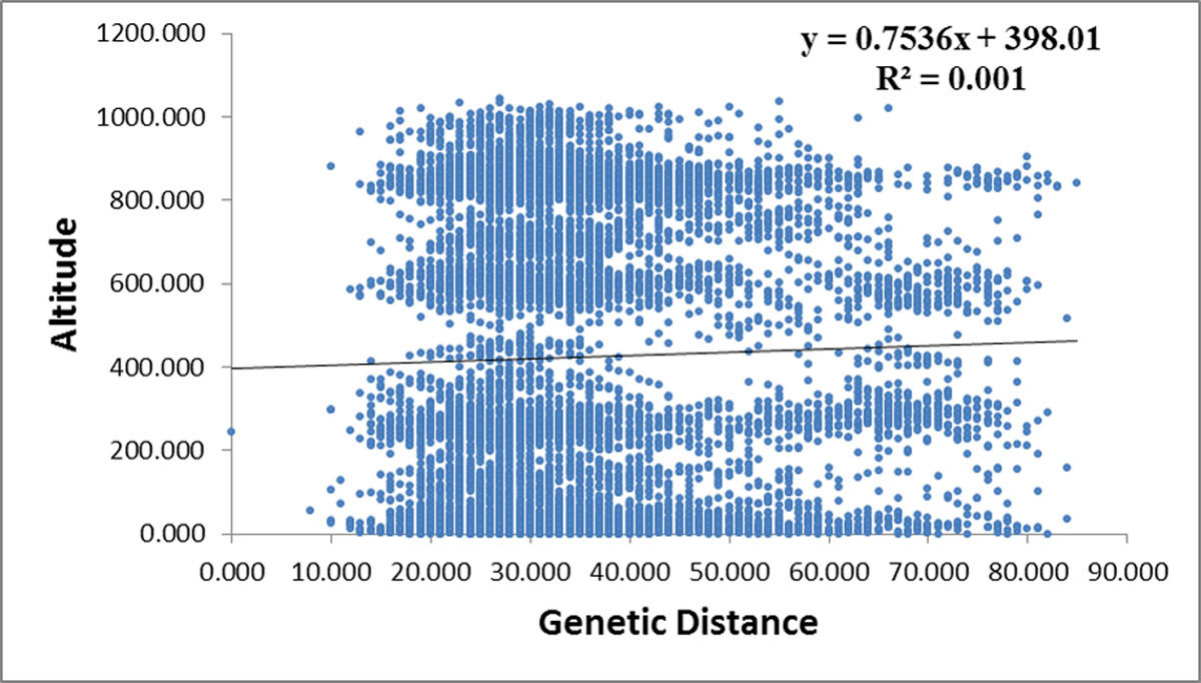
**Relevance between genetic distance and altitude**.

The SSR data was sorted in order of altitude. Population structure analysis was processed according to the known order of individuals, yielding an optimal of K = 2 [34]. Estimated populations of the 172 individuals are shown (Figure 3). The samples plot showed that low- and high-altitude individuals were considered to originate from a single group. However, the medium-altitude group was an admixture of the high- and low-altitude groups, and there was no clear separation between the groups (Figure 4). The *F*_ST _value showed little differentiation between the populations. Since STRUCTURE could not perform an analysis of K = 1 on populations with no difference, we did not accept the results of K = 2, based on the low *F*_ST _and the genetic parameters pattern among the three populations. To compare the structure of new clusters (cluster 1 and cluster 2), further STRUCTURE analyses were performed in cluster 1, which contained individuals from a high altitude, and cluster 2 from low altitude. It shows no clear structure despite peaks in K = 3 for cluster 1 and K = 2 for cluster 2 (Figure 5). The ancestry values of all of the individuals revealed that each had an equal probability of being grouped in cluster 1, 2 or 3 for the high- and low-altitude clusters. An analysis based on the Mantel test (Figure 6) showed that genetic distance was not significantly correlated with altitude (r^2 ^= 0.001, p ≤ 0.07), suggesting that altitude was not the principal factor influencing genetic differentiation in the Tibetan poplar.

## Discussion

### SSR markers and neutrality

In this study, we used GeneMarker version 1.75 to identify the fluorescently labeled PCR products. We selected 24 SSR primer pairs based on the *Populus euphratica *genome, to analyze genetic diversity within three populations of Tibetan poplar living at different altitudes in Linzhi, Tibet. As the loci were transferable between the two species, this indicates that they may be sited in a conserved region. However, based on the *F*_ST _using LOSITAN, one locus appeared to be an outlier. SSR loci mutations occasionally occur as a result of the stress of adapting to a change of environment [36] or an external stimulus [37]. Further, studies have shown that some SSR loci are non-neutral [38,39], and for this reason it is essential that a neutrality test is performed before the SSR data are used in any further analysis. The outlier locus sequence was processed using NCBI BLAST [35], and indicated high homology with a protein in *Populus trichocarpa*. We conclude that the microsatellite may be linked to expressed genes, and therefore, neutrality should not be assumed, but tested in all of the markers before genetic diversity and structure analysis. This type of marker, however, could be useful for phylogenetic studies of closely related species [40,41].

### Genetic diversity

As expected from perennial and woody species ranging across most areas of the Qinghai-Tibet plateau, the study population contained a high level of genetic diversity, but we did not identify any significant differences among the three populations from different altitudes. The number of alleles per locus in our study was less than in other related *Populus *species [42]. A mean of 6.1 alleles per locus was identified from the existing literature on *Populus *genetic diversity [42]. The *N*a of 3.73 in our study is lower than the *N*a in *P. tremuloides *(4.9) as described previously [43]. The difference is most likely due to the limited sampling area. We only collected samples from one mountain area, whereas the Tibetan poplar is distributed throughout southwestern China, of which our samples were from a limited proportion, as we aimed to study adaptation and genetic diversity along an altitude gradient. The samples from high, medium, and low altitudes appeared to be similar in genetic diversity and showed no evidence of local adaptation in the study area. STRUCTURE analysis showed that the population could be divided into two groups (clusters), with individuals from the lower altitude clustered into group 1, and those from the higher altitude clustered into group 2. Altitude appeared to have a direct relationship with the distribution of the groups, but the *F*_ST _value showed little differentiation between the populations. As STRUCTURE could not provide data for K = 1, we rejected the result showing that the population was divided into two groups. There was no peak in the estimate of the log-likelihood of the cluster number (*L*(*k*)) since the lowest likelihood was for K = 1, and *L*(*k*) either consistently increased or showed an erratic pattern with increasing variance, with all individuals admixed and the proportion of any individual assigned to each subpopulation remaining roughly similar. The Evanno criterion, ΔK [34], was not relevant as it can only be computed for K ≥ 2 and does not enable comparison of results from K = 1. For K > 2, the value of ΔK remained close to 0 in this study.

The population structure and Mantel test results suggest that the relationship between genetic diversity and altitude is not significant, and hence it is possible to hypothesize that the species has not had sufficient time for evolutionary differentiation to occur along an altitude gradient.

### Low F_ST _and strong gene flow

*F*_ST _was low for all loci, except for SSR 18. There was no noticeable differentiation among populations at three different altitudes. This may contribute to the local geographic structure and strong gene flow among individuals. The STRUCTURE results showed that the medium-altitude group was an admixture of the low- and high-altitude groups, clearly indicating that the mountain harbored two groups (clusters) of poplars, and that they separated into these clusters at an altitude of ~2700 m. Because the study area altitude ranged from 2 000 to 4 000 m, and the Tibetan poplar is distributed from 2000 to 3096 m, the tree line represented a limiting factor for tree distribution, but it appeared to have had limited impact on gene exchange between individuals and did not hinder pollen or seed dispersal. In this study, gene flow occurred among the populations. Gene flow is a vital element in local adaptation studies, because it can instruct the establishment of the local genetic structure or influence it indirectly. Gene flow among populations can also lead to combining of gene pools, reducing genetic variation among groups [44]. Therefore, gene flow acts strongly against speciation in evolutionary processes [45], by recombining the gene pools of the groups. Gene flow plays a part in evolution through pollen dispersal, seed dispersal, and the establishment of the individual adult. A geographic barrier increases the probability of extinction or local adaptation of a population, as it may push the population to evolve into a different population with a unique genetic structure, or even into a new species [46,47]. However, gene flow could also be a constraining force of natural evolution by homogenizing populations under a heterogeneous environment, and balancing gene distribution and spread [48]. However, gene flow can also be considered a creative force in evolution, where superior genes or combinations of genes are spread by gene flow [49,50]. For local adaptation, gene flow and selection are usually considered as the main forces affecting the processes of establishment. This is especially true for high outcrossing trees and perennial species, where there is extensive gene flow [51]. In summary, the factors contributing to the low level of differentiation among populations at different altitudes include: (1) Pollen dispersal and an overlapping flowering period of all three populations (high, medium and low altitude). Generally the flowering phase of *Populus *is of long duration; for example, flowering in *P. × canadensis *and *P. nigra *[52] lasts for 15 and 31 days, respectively. (2) Seed dispersal mechanisms. Most *Populus *trees live adjacent to rivers and roads, and some in the river channel itself. Therefore, rivers cannot be ignored as an important factor in seed dispersal. Poplar populations are evolutionarily homogeneous. The germplasm and genetic diversity of the Tibetan poplar could be protected by random selection in the future work, which couldprovid all of the genetic diversity to date. An unpublished experiment comparing poplars at two sites showed some differences in the growth rate, leaf characteristics, and branch numbers, etc. of individual clones sampled at different altitudes, indicating that natural selection conserved some fitness types. Genes linked to adaptation mechanisms could contribute to phenotypic variation without genetic structure differentiation which has been proved in this study. Consequently, this makes the population ideal for identifying functional genes and mechanisms of adaptation to high altitudes.

## Conclusion

To our knowledge, this is the first genetic analysis of the Tibetan poplar. The results indicate that the Tibetan poplar populations living at different altitudes on the Sejila mountain have a low level of differentiation. They have an excellent ability to adapt to different altitudes; however, local adaptation is not observed due to the lack of a geographic barrier. The high levels of gene flow lead to a low *F*_ST_, as was observed. We consider the Sejila mountain population to be appropriate for investigation of the mechanisms of adaptation to high altitudes, despite the low level of genetic structure differentiation among populations at different altitudes.

## Competing interests

The authors declare that they have no competing interests.

## Authors' contributions

Rongling Wu designed the study. Dengfeng Shen, Wenhao Bo and Fang Xu, contributed extensively to the samples collection. Dengfeng Shen performed PCR experiments and genetic diversity analysis. Dengfeng Shen and Rongling Wu wrote the manuscript. Wenhao Bo and Fang Xu prepared and revised the manuscript. All authors read and approved the final manuscript.

## Declarations

Publication charges for this article came from the Special Fund for Forest Scientific Research in the Public Welfare (201404102), NSF/IOS-0923975, Changjiang Scholars Award and "Thousand-person Plan" Award.

This article has been published as part of *BMC Genetics *Volume 15 Supplement 1, 2014: Selected articles from the International Symposium on Quantitative Genetics and Genomics of Woody Plants. The full contents of the supplement are available online at http://www.biomedcentral.com/bmcgenet/supplements/15/S1.
